# Ruelle Zeta Function from Field Theory

**DOI:** 10.1007/s00023-020-00964-8

**Published:** 2020-10-06

**Authors:** Charles Hadfield, Santosh Kandel, Michele Schiavina

**Affiliations:** 1 IBM T.J. Watson Research Center, 1101 Kitchawan Rd, Yorktown Heights, NY 10598 USA; 2 Mathematics Institute, University of Freiburg, 79104 Freiburg, Germany; 3 Department of Mathematics, ETH Zürich, Rämistrasse 101, 8092 Zurich, Switzerland; 4 Institute for Theoretical Physics, ETH Zürich, Wolfgang-Pauli strasse 27, 8093 Zurich, Switzerland

## Abstract

We propose a field-theoretic interpretation of Ruelle zeta function and show how it can be seen as the partition function for *BF* theory when an unusual gauge-fixing condition on contact manifolds is imposed. This suggests an alternative rephrasing of a conjecture due to Fried on the equivalence between Ruelle zeta function and analytic torsion, in terms of homotopies of Lagrangian submanifolds.

## Introduction

Quantum field theory is a useful tool in many areas of pure and applied mathematics. It provides a number of precise answers, often involving insight coming from statements that are theorems in finite dimensions, and that need to be appropriately checked and generalised in infinite dimensions.

A positive example of this is the interpretation by Schwarz of the Ray–Singer analytic torsion in terms of a partition function for a degenerate functional [56, 57]. The main ingredient in Schwarz’s construction is a topological field theory involving differential forms, which enjoys a symmetry given by the shift of closed forms by exact ones [8, 19, 28]. This is known nowadays with the name of *BF* theory.

From a field-theoretic point of view, such symmetry needs to be removed, or gauge fixed, as it represents a fundamental redundancy in the description. One possible way to do this is by choosing a reference metric *g* and enforcing a *g*-dependent condition on fields.^1^ It allows to compute the partition function of the theory—the starting point for quantum considerations on the system—and one is left to show that the choice of metric is immaterial. The proof that such choice of metric is irrelevant was given by Schwarz for the partition function of abelian *BF* theory, and it is tantamount to the statement of independence of the analytic torsion on the metric used to define a Laplacian on the underlying manifold.

There are several ways of encoding a choice of gauge fixing within the framework of field theory, starting from the original idea of Faddeev and Popov [35], later understood in terms of Lie algebra cohomology by Becchi, Rouet, Stora and Tyutin [13–15, 61]. A more general approach follows the ideas of by Batalin and Vilkovisky [16, 17] and implements the choice of a gauge as the choice of a Lagrangian submanifold in an appropriate (graded)-symplectic manifold of fields ${\mathcal {F}}$. In this context, gauge-fixing independence is phrased in terms of isotopies of embedded Lagrangian submanifolds and needs to be proved in some appropriate regularisation scheme. In finite dimensions, this is a theorem: the partition function for an action functional *S* that satisfies the quantum master equation (a differential condition on *S*) does not depend on the choice of a particular Lagrangian submanifold inside a smooth family $\mathbb {L}_t\subset {\mathcal {F}}$. Observe that this statement can be phrased as local constancy of the partition function w.r.t a parametrisation of the Lagrangian homotopy.

Leaving field theory aside for a moment, consider a closed manifold *M* endowed with an Anosov vector field (Definition 15). The flow associated with the vector field is a typical example of a dynamical system displaying “hard chaos” [43]. An important example of such a dynamical system is obtained from a Riemannian manifold $(\Sigma , g)$ whose sectional curvature is negative; then, $M=S^*_g\Sigma$, the unit cotangent bundle of $\Sigma$ (a sphere bundle), is such that the Reeb vector field *X* associated with the natural contact structure is an Anosov vector field and its flow coincides with the geodesic flow.

If an Anosov flow (generated by the vector field *X*) admits closed orbits, one defines a (dynamical) Ruelle zeta function $\zeta _X(\lambda )$ to count lengths of closed orbits associated with the flow in a similar spirit to how the Riemann zeta function counts prime numbers [54, 55]. The zeta function also may be defined in the presence of a representation $\rho$ of $\pi _1(M)$ which provides a flat vector bundle over *M*; this leads to a twisted zeta function. The chaotic nature of the dynamical system ensures that the zeta function is well defined for ${\text {Re}}(\lambda )\gg 1$; however, some work is required to show that the function extends meromorphically to the whole complex plane [41]. Conjecture 21, due to Fried [37], proposes that when $M=S^*_g\Sigma$, the Ruelle zeta function (evaluated at zero) exactly computes the analytic torsion of the associated sphere bundle. To connect this to field theory, we observe that this means Ruelle zeta function is expected to compute—in Schwarz’s terms—the partition function of *BF* theory in a given (metric dependent) gauge fixing.

Fried’s conjecture has received considerable attention recently. The proposed equality was confirmed in [37] for $\Sigma$ a hyperbolic manifold and conjectured in [38] that it also holds for compact locally symmetric spaces with nonpositive curvature. Conjecture 21, as we state it, appears in [39]. A more precise version for locally symmetric manifolds has been proved in [58] following [52]. In the variable curvature case, a perturbative result has been obtained in [32] and extended in [20]. A surprising result in the case of surfaces with variable negative curvature, but without reference to an acyclic representation, showed the zeta function at zero is determined by the topology of the surface [34]. This has been extended to the case of surfaces with boundary [45] and to higher-dimensional closed manifolds perturbatively close to hyperbolic space [47]. However, Fried’s conjecture, along with its three star bounty [65, Section 3, footnote 6], remains open.

### What to Expect from This Paper

We present a new class of gauge fixings for *BF* theory on contact manifolds based on the Reeb vector field associated with the contact structure; we call this the contact gauge in Definition 34. We then go on to show that, on sphere bundles with an Anosov–Reeb vector field, the Ruelle zeta function can be interpreted as an appropriately regularised determinant for the Lie derivative operator ${\mathcal {L}}_X$ on *k*-forms in the kernel of the contraction $\iota _X$. Taking this regularised determinant as the definition for the partition function of *BF* theory in the contact gauge allows us to conclude that this coincides with the Ruelle zeta function. This point of view is analogous to Schwarz’s calculation of the partition function of *BF* theory (in the metric gauge), whose output is the analytic torsion, and to the recent proof of Chern–Gauss–Bonnet theorem that has been given with similar techniques in [9] (see also [23]).

As a consequence, we relate the expected gauge-fixing independence of the partition function of *BF* theory to Fried’s conjecture; in particular, we show how modern proofs of the conjecture for certain classes of manifolds (see [32]) can be taken as a proof of gauge-fixing independence. On the other hand, we believe that the field-theoretic presentation of the Ruelle zeta function provided in this paper will allow the problem to be tackled from a different angle: by means of homotopies of Lagrangian submanifolds.

To this aim, we set up a convenient construction to compare Anosov vector fields that are related to a choice of a metric on a base manifold $\Sigma$ (Sect. 5). By means of a natural construction for sphere bundles, we map smooth paths of metrics into smooth paths of Anosov vector fields, effectively constructing an isotopy between their associated Lagrangian submanifolds. This, together with the crucial local constancy results of [32], allows us to test our approach to the known case of 2d surfaces—Theorem 47 provides an alternative proof of Fried’s conjecture on surfaces—and interprets it as gauge-fixing independence for *BF* theory.

From the point of view of algebraic topology, this result suggests that, under certain assumptions, $\iota _X$ can be made into a chain contraction for the de Rham complex; namely, one can construct $\eta _X=({\mathcal {L}}_X)^{-1} \iota _X$ with the appropriate conditions of nondegeneracy of ${\mathcal {L}}_X$. This interpretation appears to be related to the notion of a dynamical torsion introduced in [20]. There appears to be a sweet spot at the intersection of Anosov and Reeb vector fields where the independence of the “torsion” of the de Rham complex on the choice of a chain contraction and independence of the partition function of *BF* theory on a choice of gauge fixing appear to be aspects of the same statement, expressed by Fried’s conjecture.

This work is mostly addressed to the mathematical physics community working with or closely related to field theory in the Batalin–Vilkovisky formalism, but it is also aimed at the community interested in the microlocal analysis of Anosov/geodesic flows and Fried’s conjecture. Therefore, we will present some basic background on field theory with symmetries to set the stage, terminology and expectations, but we will not present a complete treatment of the mathematics behind it. Results and constructions that will be somewhat assumed in this exposition of field theory can be found, for example, in [4, 24, 30, 31].

Our main goal is to present a novel link between field theory and geometric and microlocal analysis that will hopefully allow to import techniques across research fields, and stimulate fruitful interaction between scientific communities.

In Sect. 1, we give an overview on Lagrangian field theory aimed at introducing the Batalin–Vilkovisky formalism and the problem of gauge fixing. It sets the stage for the field-theoretic interpretations that will follow.

In Sect. 2, we establishe the geometric conventions and notations, clarifying what incremental/alternative data one needs at different stages, and briefly describes the analytic torsion and Anosov dynamics.

In Sect. 3, we introduce Ruelle zeta function and its *k*-form decomposition, and state Fried’s conjecture. We interpret the zeta function as a regularised (super)determinant.

In Sect. 4, we describe a field theory called *BF* theory, and we summarise the famous interpretation (due to Schwarz) of the analytic torsion in terms of the partition function of *BF* theory and introduce a new gauge-fixing condition on contact manifolds. We show how, with that gauge fixing, the partition function of *BF* theory computes the Ruelle zeta function of the associated geodesic/Anosov flow.

Finally, in Sect. 5 we interpret Fried’s conjecture in terms of gauge-fixing independence of *BF* theory in the BV formalism and suggest a construction for sphere bundles that allows to present explicit homotopies between Lagrangian submanifolds.

## Lagrangian Field Theory, the Batalin–Vilkovisky Formalism and Regularised Determinants

In this section, we will review the basics of the Batalin–Vilkovisky (BV) formalism [16, 17] for Lagrangian field theories and how it handles gauge fixing. We will use a particular kind of regularisation based on the notion of flat traces to define determinants of operators and partition functions of quadratic functionals.

### Classical Field Theory, Symmetries and Quantisation

The standard framework for Lagrangian field theories is as follows. To a compact manifold *M*, possibly endowed with extra geometric data, like a Riemannian metric or a contact structure, we associate a space of classical fields ${F}_M$, which is usually modelled on the space of sections of some vector bundle^2^$E\rightarrow M$, together with a local functional $S_M$, called action functional. Local here means that it has the form of an integral over *M* of a density-valued functional of the fields and a finite number of jets:^3^

1 $$\begin{aligned} S_M= \int \limits _{M} L_M[\phi ,\partial ^I\phi ], \end{aligned}$$

where *I* is a finite multi-index and $L_M$ is called Lagrangian density. For simplicity, we will consider compact manifolds without boundary, although it is possible to adapt the construction to noncompact ones or manifolds with boundary (see, for example, [24, 36]).

The dynamical content of the theory is encoded in the Euler–Lagrange locus ${EL}[S_M]$, the space of solution of the Euler–Lagrange equations coming from the variational problem for $S_M$. In other words, the Euler–Lagrange locus is the set of critical points of $S_M$.

The action functional might enjoy a symmetry. That is, it might be invariant under some transformation of the fields, for example when considering Lie algebra actions on fields taking values in Lie algebra modules. Symmetries are usually described by a (smooth) distribution $D_M\subset TF_M$, and they make the critical points of the action functional degenerate^4^ and will become an issue when dealing with perturbative quantisation of the theory (see below). In what follows, we will only consider symmetry distributions that are involutive.

Quantisation, loosely speaking, is meant to replace the (commutative) algebra of functions over the space of physical configurations of the system with some (noncommutative) algebra of operators over a suitable vector space, also called the space of quantum states. Without delving too much into how this is achieved in general, for our purposes it will be important to mention that one possible procedure starts by making sense of the following expression:

2 $$\begin{aligned} Z=\int \limits \mathrm {exp}\left( \frac{i}{\hbar } S_M\right) , \end{aligned}$$

usually called the partition function, where the integral sign should ideally represent actual integration over ${F}_M$, with some measure. However, an appropriate integration theory for such (infinite-dimensional) spaces of fields is generally not available, and one defines the previous expression as a formal power series expansion in the parameter $\hbar$. This approach, however, requires the critical points of $S_M$ to be isolated, as it involves a saddle point or stationary phase approximation around critical points. It therefore automatically fails in the presence of symmetries, unless appropriate prescriptions are enforced.

We choose to deal with this problem by means of the Batalin–Vilkovisky (BV) formalism.

### Cohomological Approach and the BV Complex

Degenerate functionals are usually accompanied by involutive (symmetry) distributions $D_M$, and the space of inequivalent field configurations is the quotient ${EL}[S_M]/D_M$. Most of the times the quotient is singular, and one looks for a replacement for it.

A resolution of ${EL}[S_M]/D_M$ is given by a complex $(C^\bullet ,d_C)$ such that^5^ for all $i>0$

3 $$\begin{aligned} H^{-i}(C^\bullet )=0, \qquad H^0(C^\bullet )\simeq C^\infty ({EL}[S_M]/D_M). \end{aligned}$$

One way to obtain a resolution is by first localising to the submanifold ${EL}[S_M]$ constructing the Koszul–Tate complex and then following the Chevalley–Eilenberg procedure to describe $D_M$-invariant functions on it (see [59] for a “geometric” jet-bundle explanation of this and [36] for a more “algebraic” one).

The Batalin–Vilkovisky formalism is essentially the interpretation of said complex as the space of functions over a $(-1)$-symplectic graded manifold $({\mathcal {F}}_{BV},\Omega _{BV})$ [24, 25, 29, 46, 59], whose degree-0 part coincides with the original space of fields $F_M$, endowed with an odd vector field of degree-1 $Q\in C^\infty ({\mathcal {F}}_{BV},T[1]{\mathcal {F}}_{BV})$ such that^6^$[Q,Q]=0$ and a degree-0 functional ${\mathcal {S}}_M: {\mathcal {F}}_{BV}\rightarrow {\mathbb {R}}$ satisfying

4 $$\begin{aligned} \iota _Q\iota _Q\Omega _{BV}=\{{\mathcal {S}}_M,{\mathcal {S}}_M\}_{\Omega _{BV}}=0, \end{aligned}$$

with $\{\cdot ,\cdot \}_{\Omega _{BV}}$ the Poisson bracket associated with the symplectic structure $\Omega _{BV}$, and the compatibility condition

5 $$\begin{aligned} \iota _Q\Omega _{BV} = d {\mathcal {S}}_M. \end{aligned}$$

#### Remark 1

In infinite dimensions, one models ${\mathcal {F}}_{BV}$ on some appropriate space of sections of the jet bundle of a vector bundle $E\rightarrow M$. Then, the de Rham differential in Eq. (5) is replaced with $\delta$, the variation operator interpreted as the vertical differential on local functionals over ${\mathcal {F}}_{BV}$ (see [4]). Observe that we could take Eq. (4) as a definition of Poisson brackets in infinite dimensions.

On $C^\infty ({\mathcal {F}}_{BV})$, one constructs another (second order) differential ($\Delta _{BV}^2=0$) called BV-Laplacian and defines gauge fixing to be the choice of a Lagrangian submanifold ${\mathbb {L}}\subset {\mathcal {F}}_{BV}$. The main results are as follows:

#### Theorem 2

Let $({\mathcal {F}}_{BV},\Omega _{BV})$ be a finite-dimensional $(-1)$-symplectic graded manifold, with a measure $\mu$ and the BV Laplacian $\Delta _{\mu }$, a coboundary operator defined on $C^\infty ({\mathcal {F}}_{BV})$ such that for all $f\in C^\infty ({\mathcal {F}}_{BV})$ we have

6 $$\begin{aligned} \Delta _{\mu } f = -\frac{1}{2} div_{\mu }(X_f) \end{aligned}$$

with $X_f$ the Hamiltonian vector field of *f* with respect to $\Omega _{BV}$. Assuming that $\Delta _{\mu _{BV}} f=0$ and $g=\Delta _{\mu _{BV}} h$, with $f,g,h\in C^\infty ({\mathcal {F}}_{BV})$ then:

- for any Lagrangian submanifold ${\mathbb {L}}\subset {\mathcal {F}}_{BV}$
  7 $$\begin{aligned} \int \limits _{{\mathbb {L}}} g\ \mu _{BV}\vert _{{\mathbb {L}}} =0; \end{aligned}$$
- given a continuous family of Lagrangian submanifolds ${\mathbb {L}}_t$
  8 $$\begin{aligned} \frac{d}{\mathrm{d}t}\int \limits _{{\mathbb {L}}_t} f\ \mu _{BV} \vert _{{\mathbb {L}}_t}= 0. \end{aligned}$$

#### Remark 3

The definition of the BV Laplacian in (6) implies the relations

9 $$\begin{aligned} \Delta _{\mu }(fg)&= (\Delta _{\mu }f) g + (-1)^{|f|} f (\Delta _{\mu }g) + (-1)^{|f|}\{f,g\}_{\Omega _{BV}} \end{aligned}$$

10 $$\begin{aligned} \Delta _{\mu } \{f,g\}_{\Omega _{BV}}&= \{\Delta _{\mu }f,g\}_{\Omega _{BV}} + (-1)^{|f|+1} \{f,\Delta _{\mu }g\}_{\Omega _{BV}}, \end{aligned}$$

making the tuple $\left( C^\infty ({\mathcal {F}}_{BV}),\, \cdot \, , \{\cdot ,\cdot \}_{\Omega _{BV}},\Delta _\mu \right)$ into a BV algebra [21].

#### Remark 4

Theorem 2 is stated for finite-dimensional manifolds. In this case, $\Delta _{\mu }$ always exists. On infinite-dimensional manifolds, a number of complications arise. One needs an appropriate regularisation of $\Delta _{\mu }$, and the corresponding adaptations of statements in Theorem 2 must be checked. In this paper, we are interested in abelian *BF* theory, which is a noninteracting topological field theory whose partition function (cf. Eq. (2)) is expected to be independent of the gauge fixing, and has been computed to be the Reidermeister (or equivalently) analytic torsion of the de Rham complex [56, 57]. More recent work generalised the BV theorem for certain classes of field theories (and gauge fixings) [22, 27], while a perturbative approach has been shown to work for the theory at hand in the presence of boundaries [25], and with a regularisation coming from cellular decompositions in [26].

#### Remark 5

Notice that the notion of Lagrangian submanifold has to be appropriately adapted in infinite dimensions and when dealing with ${\mathbb {Z}}$-grading. The Lagrangian submanifolds *L* we will consider in this paper are such that, locally, the symplectic space looks like $L\oplus K$, with the symplectic form given by a nondegenerate pairing between *L* and *K*. This notion of a Lagrangian submanifold coincides with the one used in [18]. Often *L* can be seen as the vanishing locus of a Poisson subalgebra ${\mathcal {I}}$ of the Poisson algebra of functions on ${\mathcal {F}}_{BF}$, which is also isotropic, i.e. $\Omega _{BF}\vert _{L} = 0$. This means that *L* is isotropic and coisotropic.^7^ For a more in-depth analysis of Lagrangian submanifolds in infinite dimensions and the symplectic category, we refer to [64], building on [63].

#### Remark 6

For concreteness, in what follows we will discuss field theories where fields are given by differential forms on a manifold. If needed, one can think of the space of fields as a Fréchet vector space, but indeed this specification will not be necessary for our purposes.

### Partition Functions

If we look at the quadratic part of the action functional, we can interpret the partition function as a (formal) Gaussian integral. We assume from now on that the action functional is at most quadratic.

In finite dimensions, the result of said integral would be the determinant of the operator featured in the action functional $S_M$. In infinite dimensions, this requires defining an appropriate regularisation of determinants.

The standard approach to partition functions for degenerate quadratic functionals follows from Schwarz [56, 57], where the resolution of (the kernel of) an elliptic differential operator on a closed Riemannian manifold is presented, which outputs a (co)chain complex, and the partition function is given in terms of products of (regularised) determinants of operators associated with the resolving complex. The explicit example for *BF* theory is given in Sect. 4.1. In short, the insights from Schwarz allow us to interpret partition functions of quadratic degenerate functionals as (a product of) regularised determinants, provided a suitable resolution can be found such that the regularised determinants of the associated operators exist.

For the purposes of this paper, instead of the standard zeta function regularisation, we will use the notion of a flat determinant, based on flat traces, inspired by Atiyah and Bott’s constructions [2, 3]. Details on the definition of flat traces and determinant can be found in several places: [17, Definition 3.12], a microlocal version in [33, Section 2.4], and a mollifier approach in [7, Section 3.2.2]. Since we will not be concerned with the microlocal analysis of operators, we will avoid discussing the necessary tools to define flat traces, and will only work up to the requirements that they exist for the operators we will consider. In this spirit, we give the following definition.

#### Definition 7

Let $A:V\rightarrow V$ be an operator on an appropriate inner product space, such that the flat trace $\mathrm {tr}^\flat (\mathrm {exp}(-t(A+\lambda )))$ exists for $\lambda \in {\mathbb {C}}$. We define the flat determinant of $A+\lambda$ to be

11 $$\begin{aligned} \log \det {}^\flat (A + \lambda ) :=- \left. \frac{d}{\mathrm{d}s}\right| _{s=0} \left[ \frac{1}{\Gamma (s)} \int \limits _0^{\infty } t^{s-1} {{\,\mathrm{tr}\,}}^\flat (\exp {(-t(A + \lambda ))} - \Pi _\lambda )\mathrm{d}t \right] \end{aligned}$$

where $\Pi _\lambda$ is the spectral projector on the kernel of $(A + \lambda )$, whenever the integrals converge.

#### Remark 8

Observe that if *A* is such that $\mathrm{e}^{-t(A + \lambda )}$ is trace class, then ${{\,\mathrm{tr}\,}}(\mathrm{e}^{-t(A + \lambda )})={{\,\mathrm{tr}\,}}^\flat (\mathrm{e}^{-t(A+\lambda )})$. As a consequence, if the zeta-regularised determinant of $A+\lambda$ exists, it coincides with the flat determinant: ${\det }^{\flat }(A+\lambda )=\det (A+\lambda )$. See, for example, [7, Proposition 6.8] for details.

Let $A=\mathrm {diag}(B,C)$ be a graded, degree-preserving (block-diagonal) linear map on a finite-dimensional graded vector space. A graded Gaussian integral for $\exp {(-\langle y,Ax\rangle })$ returns $\mathrm {sdet}(A)^{-1}=\frac{\mathrm {det}(C)}{\det (B)}$. More generally, if $A_k$ is the *k*th component of *A* acting on a graded space with a finite number of nonzero components, each $A_k$ acting on vectors of degree *k*, we get^8^

$$\begin{aligned} \mathrm {sdet}(A)=\prod _{k=0}^n \det (A)^{(-1)^k}. \end{aligned}$$

For an introduction to Berezinians and odd integration, see, for example, [51, Section 3.8] and [62], while the original notion was introduced in [10, 11].

In infinite dimensions, to an operator *A* on a graded space we can associate a regularised superdeterminant in the same way, but replacing the determinants on the block operators with their flat-regularised versions. We will use this notion to define the partition function of a degenerate quadratic functional, as follows:

#### Definition 9

Let $S:V\rightarrow {\mathbb {R}}$ of the form $S= \frac{1}{2}\int _M (x,Ax)$ for some operator $A:V\rightarrow V$, with *V* a (possibly graded) vector space endowed with an inner product $(\cdot ,\cdot )$. We define the partition function *Z* of *S* to be the square root of the flat (super)determinant of the (graded) operator *A*:

12 $$\begin{aligned} Z(S)= \int \limits _{V} \mathrm{e}^{i S_M} := |{\mathrm {sdet}}^\flat (A)|^{-\frac{1}{2}}. \end{aligned}$$

When the field theory has symmetries, we assume that *V* is further endowed with a $(-1)$ symplectic form. A choice of a Lagrangian submanifold ${\mathbb {L}}\subset V$ will be called gauge fixing, and we define the partition function of *S* in the gauge fixing ${\mathbb {L}}$ to be

13 $$\begin{aligned} Z(S,{\mathbb {L}}):=Z(S\vert _{{\mathbb {L}}}). \end{aligned}$$

#### Remark 10

To obtain a true generalisation of finite-dimensional Gaussian integrals, one should append to formula (12) the phase factor $\exp (-i\frac{\pi }{4}\mathrm {sg}(A))$ where $\mathrm {sg}(A)$ is the signature of the operator *A*, appropriately regularised. Since in what follows we will not discuss the phase of partition functions, we will omit this term from the definition.

#### Remark 11

Observe that often one encounters the situation in which $S_M=\int _M (y,Bx)$ for some operator *B* and $x,y\in V'$ for some space $V'$. Define^9^$V=V'\oplus V'$ and

$$\begin{aligned} A= \left( \begin{array}{cc} 0 &{} B \\ B^t &{} 0 \end{array}\right) \end{aligned}$$

so that $(y,Bx) = \frac{1}{2} (z,Az)$ with $z= (x,y) \in V$. Then $Z=|\mathrm {sdet}^\flat (A)|^{-\frac{1}{2}} = |\mathrm {sdet}^\flat (B)|^{-1}$.

#### Remark 12

Let *V* be a graded vector space and *V*[*k*] its *k*-shift, so that $(V[k])^i := V^{i+k}$. In particular, if $z\in V[1]$ is a homogeneous element of degree *k* it will be parametrised by homogeneous elements in *V* of opposite parity. In particular, if *A* is a graded linear map on *V*, the Gaussian integral

14 $$\begin{aligned} \int _{V[1]} \mathrm{e}^{-\frac{1}{2}(z,A,z)} := |{\mathrm {sdet}}^\flat (A)|^{\frac{1}{2}}. \end{aligned}$$

## Geometric Setting

This section establishes the geometry and notations. In the following three sections, we progressively introduce more structure to our initial set-up of a flat vector bundle over a manifold, whose twisted cohomology is trivial. The plainest setting involves a differentiable manifold endowed with a flat vector bundle. On top of that, we consider the introduction of either a Riemannian structure or a contact structure. The intersection of the two will require the base manifold to display Anosov dynamics.

It is useful to distinguish between these geometric settings as when we will only need certain geometric properties when discussing different field theories. This distinction between geometric data with which a differentiable manifold is endowed reflects the practice of complementing topological theories with additional geometric structures (e.g. Riemannian or contact) for the sake of gauge fixing.

### Flat Vector Bundle

Let *M* be an *N*-dimensional compact manifold without boundary which is oriented and connected. Let $\rho :\pi _1(M)\rightarrow U(\mathbb {C}^r)$ denote a unitary representation. This representation endows *M* with a Hermitian vector bundle (*E*, *h*) of rank *r* with flat connection $\nabla$. We collectively denote these data by (*M*, *E*).

Let $\Omega ^\bullet (M;E)$ denote the space of (smooth) differential forms on *M* taking values in *E*, and let

15 $$\begin{aligned} d_\nabla \equiv d_k :\Omega ^k(M,E) \rightarrow \Omega ^{k+1}(M,E) \end{aligned}$$

be two notations for the twisted de Rham differential. We denote by $H^\bullet (M;E)$ the cohomology associated with the twisted de Rham complex $(\Omega ^\bullet (M;E), d_\nabla )$, with Betti numbers $\beta _k:= \dim H^k(M;E)$.

From now on, we will assume that (*M*, *E*) is such that its twisted de Rham complex is acyclic, i.e. $\beta _k=0$ for all $0\le k \le N$.

### Riemannian Structure and Analytic Torsion

Suppose (*M*, *E*) is supplemented with a Riemannian metric $g_M$. We collectively denote these data $(M,E,g_M)$. Let $\mathrm {dVol}$ denote the associated volume form and let $\langle \cdot , \cdot \rangle$ be the inherited inner product on $\bigwedge ^\bullet T^*M$. The Hodge star $\star : \bigwedge ^k T^*M \rightarrow \bigwedge ^{N-k}T^*M$ is defined through

16 $$\begin{aligned} u \wedge \star v = \langle u, v \rangle \mathrm {dVol}\end{aligned}$$

for $u,v\in \bigwedge ^k T^*M$. This lifts to *E*-valued forms by identifying *E* with its dual via the Hermitian metric *h*. An inner product is then placed on $\Omega ^\bullet (M;E)$ by declaring

17 $$\begin{aligned} (u,v)=\int _{M}[u\wedge \star v]^{\mathrm {top}} \end{aligned}$$

for $u,v\in \Omega ^\bullet (M;E)$ with $\mathrm {top}$ refers to only taking the top-form (degree *N*) part of $u\wedge \star v$.

The de Rham differential has an adjoint

18 $$\begin{aligned} d_\nabla ^*\equiv d_k^* :\Omega ^{k+1}(M,E) \rightarrow \Omega ^k(M,E) \end{aligned}$$

which provides the twisted Laplace–de Rham operator (henceforth simply Laplacian)

19 $$\begin{aligned} \Delta _k := (d_\nabla ^* + d_\nabla )^2 : \Omega ^k(M;E) \rightarrow \Omega ^k(M;E). \end{aligned}$$

When acting on $L^2(M;\bigwedge ^k T^*M \otimes E)$, the Laplacian has nonnegative eigenvalues $\lambda _n\ge 0$. Denote by $\Pi _{\lambda }$ the $L^2$ spectral projector onto the kernel of $\Delta _k+\lambda$, and consider the function

20 $$\begin{aligned} F_{\Delta _k} (\lambda ,s)&:= \frac{1}{\Gamma (s)} \int _0^\infty t^{s-1} {{{\,\mathrm{tr}\,}}}^\flat \left( \mathrm{e}^{-t(\Delta _k+\lambda )} - \Pi _{\lambda } \right) \mathrm{d}t. \end{aligned}$$

The (flat)-regularised determinant of the operator $\Delta _k + \lambda$ is

21 $$\begin{aligned} \log {\det }^\flat (\Delta _k + \lambda ) = - \left. \frac{\partial }{\partial s}\right| _{s=0} F_{\Delta _k}(\lambda , s). \end{aligned}$$

We also write $f_{\Delta _k}(s) := F_{\Delta _k}(0,s)$ and observe that $f_{\Delta _k}(s) = \sum _{\lambda _j>0} {\lambda _j}^{-s}$.

#### Definition 13

The analytic torsion of *M* [53] is defined to be

22 $$\begin{aligned} \tau _{\rho }(M) := \prod _{k=1}^N {\det }^\flat (\Delta _k)^{\frac{k}{2}(-1)^{k+1}}. \end{aligned}$$

Alternatively, we can write

23 $$\begin{aligned} 2\log \tau _{\rho }(M) = \sum _{k=1}^{N} (-1)^{k} k \left. \frac{d}{\mathrm{d}s}\right| _{s=0} f_{\Delta _k}(s). \end{aligned}$$

The Hodge decomposition provides an orthogonal decomposition of $L^2(M;\bigwedge ^\bullet T^*M\otimes E)$ into exact and coexact forms. (No harmonic forms are present due to acyclicity.) Introducing $d_k^*d_k:=\Delta _k|_{\text {coexact}}$, we can also write the analytic torsion as [57] (see also [26, 50])

24 $$\begin{aligned} \tau _{\rho }(M) = \prod _{k=0}^{N-1} {\det }^\flat (d_k^*d_k)^{\frac{1}{2}(-1)^{k}}, \end{aligned}$$

which alternatively reads

25 $$\begin{aligned} 2\log \tau _{\rho }(M) = \sum _{k=0}^{N-1} (-1)^{k+1} \left. \frac{d}{\mathrm{d}s}\right| _{s=0} f_{d_k^*d_k} (s). \end{aligned}$$

### Contact Structure

Suppose that (*M*, *E*) is supplemented with a contact form $\alpha \in \Omega ^1(M)$, and $\mathrm {dim}(M)= N =2n+1$. We will denote $\mathrm {dVol}= \alpha \wedge (\mathrm{d}\alpha )^n$. Let *X* be the associated Reeb vector field, defined by the relations

26 $$\begin{aligned} \iota _X\alpha =1, \qquad \iota _X \mathrm{d}\alpha =0. \end{aligned}$$

We collectively denote these data (*M*, *E*, *X*).

Denote by $T^*_0M$ the 2*n*-rank subbundle of $T^*M$ defined as the conormal of *X*, so that pointwise $T^*M = \mathbb {R}\alpha + T^*_0M$. Transferring this to the space of *E*-valued differential forms, we write

27 $$\begin{aligned} \Omega ^k(M,E) = \Omega ^k_0(M,E) \oplus \alpha \wedge \Omega ^{k-1}_0(M,E), \end{aligned}$$

where $$\Omega ^k_0(M,E)=\ker \iota _X |_{\Omega ^k(M)}$$.

#### Contact Riemannian Structure

Let (*M*, *E*, *X*) as above and introduce a metric $g_M$ on *M* of the form $g_M=\alpha ^2+g_0$ such that $T^*M = \mathbb {R}\alpha + T^*_0M$ becomes an orthogonal decomposition. Let $\star$ and $\star _0$ denote the Hodge stars associated with $(T^*M,g_M)$ and $(T^*_0M,g_0)$, respectively. We may choose $g_0$ such that $\star \alpha =(\mathrm{d}\alpha )^n=\star _0 1$ whence the Hodge star behaves nicely with respect to the splitting of $T^*M$. Specifically, we have maps

28a $$\begin{aligned} \star : \Omega ^{k}_0(M,E)&\rightarrow \alpha \wedge \Omega ^{N-k-1}_0(M,E)&\star : \alpha \wedge \Omega ^{k}_0(M,E)&\rightarrow \Omega ^{N-k-1}_0(M,E) \end{aligned}$$

28b $$\begin{aligned} \varphi&\mapsto (-1)^{k}\alpha \wedge \star _0\varphi&\alpha \wedge \psi&\mapsto \star _0 \psi \end{aligned}$$

Moreover, noting that $\langle \alpha \wedge \varphi , \alpha \wedge \varphi \rangle = \langle \varphi , \varphi \rangle$ on $\Omega ^{\bullet }_0(M,E)$ we conclude $(\iota _X)^T = \alpha \wedge$. When a metric is chosen in this way, compatible with the contact structure, we collectively denote these data $(M,E,X,g_M)$.

##### Definition 14

Considering the maps

29 $$\begin{aligned} \alpha \wedge :\Omega ^{\bullet }_0(M,E) \rightarrow \alpha \wedge \Omega ^{\bullet }_0(M,E),&\iota _X: \alpha \wedge \Omega ^{\bullet }_0(M,E) \rightarrow \Omega ^{\bullet }_0(M,E), \end{aligned}$$

we set

30 $$\begin{aligned} {\mathrm {sdet}}^\flat (\alpha \wedge ) = {\mathrm {sdet}}^\flat (\iota _X):= \left| {\mathrm {sdet}}^{\flat }(\iota _X\circ \alpha \wedge )^{\frac{1}{2}}\right| = 1. \end{aligned}$$

### Anosov Dynamics

Suppose *M* is supplemented with a flow $\varphi _t:M\rightarrow M$ for $t\in \mathbb {R}$. We will reuse $X\in C^\infty (M;TM)$ to denote the vector field which generates $\varphi _t$.

#### Definition 15

The flow is Anosov if there exists a $\mathrm{d}\varphi _t$-invariant continuous splitting of the tangent bundle:

31 $$\begin{aligned} T_xM = E_n(x) \oplus E_s(x) \oplus E_u(x),&E_n(x)=\mathbb {R}X_x, \end{aligned}$$

and for a given norm $\Vert \cdot \Vert$ on *TM*, there exist constants $C, \lambda >0$ so that for all $t\ge 0$,

32 $$\begin{aligned}&\forall v \in E_s(x), \quad \Vert \mathrm{d}\varphi _t(x)v\Vert \le C \mathrm{e}^{-\lambda t}\Vert v\Vert , \qquad \nonumber \\&\quad \forall v \in E_u(x), \quad \Vert \mathrm{d}\varphi _{-t}(x)v\Vert \le C \mathrm{e}^{-\lambda t}\Vert v\Vert . \end{aligned}$$

The subbundles $E_n, E_s$ and $E_u$ are, respectively, called neutral, stable and unstable.

#### Remark 16

We will prefer to work with the cotangent bundle $T^*M$ due to the Lie derivative acting naturally on differential forms. The cotangent bundle also has a decomposition $T^*_xM=E_n^*(x) \oplus E_s^*(x) \oplus E_u^*(x)$ whose stable and unstable bundles are understood through the action of $\left( \mathrm{d}\varphi _{-t}\right) ^T$ (rather than $\mathrm{d}\varphi _t$).

Henceforth, we will always assume that the stable and unstable bundles are orientable and each have rank *n*.

#### A Guiding Example

We provide an example of the geometric setting discussed in the previous subsections. Let $(\Sigma , g)$ be a compact manifold without boundary which is oriented, connected and of dimension $n+1$. Suppose that $\Sigma$ has sectional curvature which is everywhere strictly negative. Let $M:=S^*_g\Sigma$ be the unit cotangent bundle of $\Sigma$. Set $\alpha \in \Omega ^1(M)$ to be the pullback of the canonical one form on $T^*\Sigma$. Then $(M,\alpha )$ is a contact manifold, and the Reeb vector field $X_g\in C^\infty (M;TM)$ generates the geodesic flow $\varphi _t$ which is Anosov [1, 5, 6].

If we consider $M=S_g^*\Sigma$ within the geometric setting (*M*, *E*) of Sect. 2.1 (in particular the representation $\rho$ is unitary and $\nabla$ is flat), we denote the resulting contact, Anosov, Riemannian data on $S_g^*\Sigma$ by

$$\begin{aligned} (M=S_g^*\Sigma , E, X_g, g). \end{aligned}$$

##### Remark 17

$M\rightarrow \Sigma$ is an $\mathbb {S}^{n}$-bundle, and if $n\ge 2$, then $\pi _1(\mathbb {S}^{n})=0$ whence representations of $\pi _1(\Sigma )$ are in one-to-one correspondence with representations of $\pi _1(M)$.

##### Remark 18

Observe that in the case of a unitary representation and a flat vector bundle, and when $n\ge 2$, the preceding remark implies that $\tau _\rho (M)=\left( \tau _\rho (\Sigma )\right) ^2$ [37, Section 1, p. 526]. For the rest of this article, particularly the announcements of theorems and conjectures, we have chosen to emphasise the role of $M=S^*_g\Sigma$ rather than $\Sigma$.

## Ruelle Zeta Function

Consider the geometric data (*M*, *E*, *X*) of Sect. 2.3 and assume the flow $\varphi _t$ associated with *X* is Anosov (see Sect. 2.4). We denote by ${\mathcal {P}}$ the set of primitive orbits of the flow $\varphi _t$ and by $\ell (\gamma )$ the period of any given $\gamma \in {\mathcal {P}}$.

### Definition 19

The Ruelle zeta function (associated with the trivial representation of $\pi _1(M)$) is defined as

33 $$\begin{aligned} \zeta (\lambda ) := \prod _{\gamma \in {\mathcal {P}}} (1 - \mathrm{e}^{-\lambda \ell (\gamma )}) \end{aligned}$$

whose convergence is assured for ${\text {Re}}\lambda \gg 1$. The Ruelle zeta function twisted by an arbitrary representation $\rho$ is

34 $$\begin{aligned} \zeta _\rho (\lambda ) := \prod _{\gamma \in {\mathcal {P}}} \det (I - \rho ([\gamma ])\mathrm{e}^{-\lambda \ell (\gamma )}), \end{aligned}$$

whose convergence is again assured for ${\text {Re}}\lambda \gg 1$.

Here, $[\gamma ]$ represents the conjugacy class of $\gamma$ in $\pi _1(M)$. It has been shown that the zeta functions continue meromorphically to $\mathbb {C}$ [12, 33, 41, 48]. We have the following:

### Theorem 20

([37]). Let (*M*, *E*, *X*, *g*) be the geometric data of Sect. 2.4.1, where $M=S^*_g\Sigma$, with $\Sigma$ a closed, oriented hyperbolic manifold $\Sigma =\Gamma \backslash {\mathbb {H}}^{n+1}$, and *g* the induced hyperbolic metric. Then, the Ruelle zeta function, defined for $\mathrm {Re}(s)>n$ by Eq. (34), extends meromorphically to ${\mathbb {C}}$ and

$$\begin{aligned} {|\zeta _\rho (0)|^{(-1)^{n}} =\tau _\rho (M).} \end{aligned}$$

This fact has inspired a conjecture [38]:

### Conjecture 21

(Fried). Let (*M*, *E*, *X*, *g*) be the geometric data of Sect. 2.4.1 with $\mathrm {dim}(M)=2n+1$. Then the (twisted) Ruelle zeta function computes the analytic torsion:

35 $$\begin{aligned} |\zeta _\rho (0)| ^{(-1)^{n}} = \tau _\rho (M). \end{aligned}$$

### Differential Forms Decomposition

This section shows how the (twisted) Ruelle zeta function may be written as an alternating product of zeta functions associated with $\left( \bigwedge ^k T^*_0M\right) \otimes E$ for $0\le k \le 2n$.

Given a closed orbit $\gamma$, of length $\ell (\gamma )$ and a point $x\in M$ in the orbit, let $P(\gamma ,x)$ denote the linearised Poincaré map on the fibre of $T^*_0M$ above *x*:

36 $$\begin{aligned} P(\gamma ,p) := \left( \mathrm{d}\varphi _{-\ell (\gamma )} \right) ^T : T^*_0M_{(x)} \rightarrow T^*_0M_{(x)}. \end{aligned}$$

This map is conjugate to $P(\gamma , x')$ for other $x'$ in the same orbit $\gamma$, and as we need only evaluate this map’s trace and determinant, we will refer to all maps as $P(\gamma )$. Recall $T^*_0M$ is the subbundle of $T^*M$ conormal to the vector field *X*.

We start with the basic linear algebra identity

$$\begin{aligned} \det (I-A) = \sum _{k=0}^{m} (-1)^k {{\,\mathrm{tr}\,}}(\wedge ^k A) \end{aligned}$$

for an endomorphism *A* on an *m*-dimensional vector space and the observation that $P(\gamma )$ has precisely *n* eigenvalues greater than 1 (where *n* is the rank of $E_u^*$). Therefore, for $j\ge 1$

37 $$\begin{aligned} (-1)^n | \det (I - P(\gamma )^j) | = \sum _{k=0}^{2n} (-1)^k {{\,\mathrm{tr}\,}}(\wedge ^k P(\gamma )^j). \end{aligned}$$

Using identity (37) to obtain equality (38c), we derive

38a $$\begin{aligned} \log \zeta _\rho (\lambda )&= \sum _{\gamma \in {\mathcal {P}}} {{\,\mathrm{tr}\,}}\log (I - \rho ([\gamma ])\mathrm{e}^{-\lambda \ell (\gamma )}) \end{aligned}$$

38b $$\begin{aligned}&= -\sum _{\gamma \in {\mathcal {P}}} {{\,\mathrm{tr}\,}}\sum _{j=1}^\infty \frac{1}{j} \mathrm{e}^{-\lambda j \ell (\gamma )} \rho ([\gamma ])^j \end{aligned}$$

38c $$\begin{aligned}&= -\sum _{\gamma \in {\mathcal {P}}} \sum _{j=1}^\infty \frac{1}{j} \mathrm{e}^{-\lambda j \ell (\gamma )} {{\,\mathrm{tr}\,}}(\rho ([\gamma ])^j) \left( (-1)^{n}\sum _{k=0}^{2n} \frac{(-1)^k {{\,\mathrm{tr}\,}}(\wedge ^k P(\gamma )^j)}{| \det (I - P(\gamma )^j) |} \right) \end{aligned}$$

38d $$\begin{aligned}&= (-1)^n\sum _{k=0}^{2n} (-1)^k \log \zeta _{\rho ,k}(\lambda ). \end{aligned}$$

Equation (38d) in the preceding display defines implicitly the Ruelle zeta function twisted by $\rho$ upon restriction to *k*-forms

39 $$\begin{aligned} \zeta _{\rho ,k}(\lambda ) := \exp \left( -\sum _{\gamma \in {\mathcal {P}}} \sum _{j=1}^\infty \frac{1}{j} \frac{ \mathrm{e}^{-\lambda j \ell (\gamma )} {{\,\mathrm{tr}\,}}(\rho ([\gamma ])^j) {{\,\mathrm{tr}\,}}(\wedge ^k P(\gamma )^j)}{| \det (I - P(\gamma )^j) |} \right) \end{aligned}$$

so in the form of a compact equality, we have

40 $$\begin{aligned} \zeta _{\rho }(\lambda )^{(-1)^n } = \prod _{k=0}^{2n} \zeta _{\rho ,k}(\lambda )^{(-1)^k}. \end{aligned}$$

### Ruelle Zeta Function as Regularised Determinant

We aim to link the function $\zeta _{\rho ,k}$ with the operator $\mathcal {L}_{X,k}$ acting on $\Omega ^k_0(M;E)$. This is done with the help of the Atiyah–Bott–Guillemin trace formula [42]. We use the notation

41 $$\begin{aligned} \mathrm{e}^{-t\mathcal {L}_{X,k}}={\varphi _{-t}}^* : L^2(M; \bigwedge ^kT^*_0M\otimes E) \rightarrow L^2(M; \bigwedge ^kT^*_0M\otimes E). \end{aligned}$$

and write the Schwartz kernel $K_k(t,\cdot ,\cdot )$ such that

42 $$\begin{aligned} (\mathrm{e}^{-t\mathcal {L}_{X,k}} \psi )(x) = \int _M K_k(t,x,y) \psi (y) \mathrm{d}y. \end{aligned}$$

Due to the microlocal structure of $K_k$ we may take its flat trace, which leads to the Atiyah–Bott–Guillemin trace formula:

43 $$\begin{aligned} {{\,\mathrm{tr}\,}}^\flat \mathrm{e}^{-t\mathcal {L}_{X,k}} = \sum _{\gamma \in {\mathcal {P}}} \sum _{j=1}^\infty \ell (\gamma ) \delta (t - j\ell (\gamma )) \frac{{{\,\mathrm{tr}\,}}(\rho ([\gamma ])^j) {{\,\mathrm{tr}\,}}(\wedge ^k P(\gamma )^j)}{| \det (I - P(\gamma )^j) |} \end{aligned}$$

as a distribution on $\mathbb {R}_+$. Integrating this distribution against the function $-t^{-1}\mathrm{e}^{-t\lambda }$ provides an integral representation of $\zeta _{\rho ,k}(\lambda )$:

44 $$\begin{aligned} \log \zeta _{\rho , k}(\lambda ) = -\int _0^\infty t^{-1}\mathrm{e}^{-t\lambda } {{\,\mathrm{tr}\,}}^\flat \mathrm{e}^{-t\mathcal {L}_{X,k}} \,\mathrm{d}t. \end{aligned}$$

Consider now the following function, dependent on two variables:

45 $$\begin{aligned} F_{\mathcal {L}_{X,k}}(\lambda ,s)&:= \frac{1}{\Gamma (s)} \int _0^\infty t^{s-1} {{\,\mathrm{tr}\,}}^\flat \mathrm{e}^{-t(\mathcal {L}_{X,k}+\lambda )} \mathrm{d}t. \end{aligned}$$

This function is holomorphic for small *s* (near $s=0$) and for ${\text {Re}}(\lambda )\gg 1$ (see Sect. 3.3). Naively, at $s=0$ the integrand poses a problem due to the $t^{-1}$ structure as $t\rightarrow 0$; however, the trace formula (Eq. (43)) provides a natural cut-off in small *t* so that we avoid this problem. Moreover, for small *s* we expand $1/\Gamma (s)=s+O(s^2)$ showing

46 $$\begin{aligned} \left. \partial _s \right| _{s=0} F_{\mathcal {L}_{X,k}}(\lambda ,s)&= \left( \left. \partial _s \right| _{s=0} \frac{1}{\Gamma (s)} \right) \cdot \left. \int _0^\infty t^{s-1} {{\,\mathrm{tr}\,}}^\flat \mathrm{e}^{-t(\mathcal {L}_{X,k}+\lambda )} \mathrm{d}t \right| _{s=0} \end{aligned}$$

47 $$\begin{aligned}&= - \log \zeta _{\rho , k}(\lambda ). \end{aligned}$$

Recalling Definition 7 for the flat determinant of an operator now indicates that, for ${\text {Re}}\lambda \gg 1$

48 $$\begin{aligned} \log {\det } ^\flat (\mathcal {L}_{X,k} + \lambda ) \equiv - \left. \partial _s \right| _{s=0} F_{\mathcal {L}_{X,k}}(\lambda ,s) = \log \zeta _{\rho , k}(\lambda ). \end{aligned}$$

Since $\zeta _{\rho ,k}(\lambda )$ has a meromorphic extension to the complex plane, so does the function $\det ^{\flat }({\mathcal {L}}_{X,k} + \lambda )$. Assuming there are no poles at $\lambda =0$, we sensibly have $\det ^{\flat }({\mathcal {L}}_{X,k})$ as the value at zero of the meromorphic extension of the Ruelle zeta function (restricted to *k*-forms) at zero:

49 $$\begin{aligned} {\det }^{\flat }({\mathcal {L}}_{X,k}) = \zeta _{\rho ,k}(0). \end{aligned}$$

The decomposition in Eq. (40) of the Ruelle zeta function now gives an operator interpretation of the Ruelle zeta function:

50 $$\begin{aligned} \zeta _\rho (\lambda )^{(-1)^n} = \prod _{k=0}^{2n} {\det }^\flat (\mathcal {L}_{X,k} + \lambda ) ^{(-1)^k}. \end{aligned}$$

#### Remark 22

Notice that, due to the decomposition (40) and the trace formula (43), we can consider $\zeta _\rho$ as directly dependent on an Anosov vector field *X*. To stress this fact, we will use the notation $\zeta (X,\lambda )$ (resp. $\zeta _k(X,\lambda )$) instead of $\zeta _\rho (\lambda )$ (resp. $\zeta _{\rho ,k}(\lambda ))$.

### Meromorphic Extension of the Resolvent

In the preceding section, we related the resolvent $(\mathcal {L}_{X,k} + \lambda )^{-1}$ to $\zeta _{\rho ,k}(\lambda )$ for $\lambda \gg 1$. Here, we announce a more precise statement for the meromorphic extension of $(\mathcal {L}_{X,k} + \lambda )^{-1}$ [33].

The operator norm of $\mathrm{e}^{-t\mathcal {L}_{X,k}}$ is bounded by $\mathrm{e}^{C_0t}$ for some $C_0>0$. Therefore, the resolvent $(\mathcal {L}_{X,k}+\lambda )^{-1}$, as an operator on $L^2$ sections, exists for ${\text {Re}}\lambda >C_0$ and is given by the formula

51 $$\begin{aligned} (\mathcal {L}_{X,k} + \lambda )^{-1} = \int _0^\infty \mathrm{e}^{-t(\mathcal {L}_{X,k} + \lambda )} \mathrm{d}t. \end{aligned}$$

The restricted resolvent

52 $$\begin{aligned} R_k(\lambda ) = (\mathcal {L}_{X,k}+\lambda )^{-1} : C^\infty (M;\bigwedge ^k T^*_0M \otimes E) \rightarrow {\mathcal {D}}'(M;\bigwedge ^k T^*_0M \otimes E) \end{aligned}$$

has a nowhere-vanishing meromorphic continuation to $\mathbb {C}$ whose poles are of finite rank, and are called Pollicott–Ruelle resonances. For each $\lambda _0\in \mathbb {C}$, we have the expansion

53 $$\begin{aligned} R_k(\lambda ) = R_k^H(\lambda ) + \sum _{j=1}^{J(\lambda _0)} \frac{(-1)^{j-1}(\mathcal {L}_{X,k}+\lambda _0)^{j-1}\Pi _{\lambda _0} }{(\lambda -\lambda _0)^j} \end{aligned}$$

where $R_k^H$ is holomorphic near $\lambda _0$ and $\Pi _{\lambda _0} : C^\infty (M; \bigwedge ^k T^*_0M \otimes E) \rightarrow {\mathcal {D}}'(M; \bigwedge ^k T^*_0M \otimes E)$ is a finite rank projector. The range of $\Pi _{\lambda _0}$ defines (generalised) resonant states. They are characterised as

54 $$\begin{aligned} {\text {Range}} \Pi _{\lambda _0} = \left\{ \varphi \in {\mathcal {D}}'\left( M,\bigwedge ^k T^*_0M \otimes E\right) : {\text {WF}}(u)\subset E^*_u, \left( \mathcal {L}_{X,k}+\lambda _0\right) ^{J(\lambda _0)}\varphi =0 \right\} , \end{aligned}$$

where ${\text {WF}}$ refers to the wave front of a distribution (or current), $E_u^*$ is the unstable bundle referenced in Remark 16 and $J(\lambda _0)$ denotes the multiplicity. The adjective “generalised” refers to the possibility that the pole may not be simple (and is superfluous in the case $J(\lambda _0)=1$).

Finally, the poles of the meromorphic continuation $R_k(\lambda )$ correspond to zeros of the zeta function $\zeta _k(\lambda )$ (which is entire for each *k*), and the rank of the projector $\Pi _{\lambda }$ equals the multiplicity of the zero.

## *BF* Theory on Contact Manifolds

In this section, we will analyse a field theory that goes under the name of *BF* theory,^10^ in the Batalin–Vilkovisky (BV) formalism. Consider the basic geometric data (*M*, *E*) of Sect. 2.1. The classical version of abelian *BF* theory (i.e. without BV Formalism) is given by the following assignment:

### Definition 23

Define the space of classical fields to be $F_{BF}:=\Omega ^1(M,E)\oplus \Omega ^{N-2}(M,E)$, and the BF action functional:

55 $$\begin{aligned} S_{BF} = \int \limits _{M} B\wedge d_\nabla A. \end{aligned}$$

Then we call *BF* theory the assignment $(M,E)\leadsto (F_{BF},S_{BF})$.

### Remark 24

Recall that Sect. 2.1 equips *E* with a Hermitian metric *h*. This metric has been implicitly used to couple *B* and *A*. Specifically, both *B* and $d_\nabla A$ take values in *E* whence *h* must be used so that $B\wedge d_\nabla A \in \Omega ^N(M)$.

### Remark 25

It is easy to see that shifting either *B* or *A* by a $d_\nabla$-exact form leaves the action functional unchanged.^11^ This goes under the name of reducible symmetry, and it is conveniently treated by means of the BV formalism.

Let us consider the space of differential forms $\Omega ^{-\bullet }(M,E)$ as a ${\mathbb {Z}}$-graded vector space, such that homogeneous forms $\omega ^{(k)}\in \Omega ^k(M,E)$ will have degree $|\omega ^{(k)}|:=-k$. We define the space of Batalin–Vilkovisky fields for *BF* theory to be the graded vector space

56 $$\begin{aligned} {\mathcal {F}}_{BF}:= \Omega ^{-\bullet }(M,E)[1]\oplus \Omega ^{-\bullet }(M,E)[N-2]\ni (\mathbb {A},\mathbb {B}) \end{aligned}$$

where the degree shift means that a *k*-form in $\Omega ^{-\bullet }(M,E)[1]$ will have degree $|\mathbb {A}|=1-k$. The symplectic structure reads

57 $$\begin{aligned} \Omega _{BF} = \int \limits _M [\delta \mathbb {B}\delta \mathbb {A}]^{\mathrm {top}}, \end{aligned}$$

and we define an action functional on ${\mathcal {F}}_{BF}$ as

58 $$\begin{aligned} {\mathbb {S}}_{BF} = \int \limits _M [\mathbb {B}d_\nabla \mathbb {A}]^{\mathrm {top}}. \end{aligned}$$

Observe that $|{\mathbb {S}}_{BF}|=0$ and $|\Omega _{BF}|=-1$.

### Remark 26

We stress that, although the functional form of $S_{BF}$ in Eq. (55) and ${\mathbb {S}}_{BF}$ in (58) is the same, in the latter $\mathbb {B}$ and $\mathbb {A}$ are inhomogeneous forms with an additional shift in degree. Such a degree shift effectively switches the total parity of inhomogeneous forms, so that, if *N* is odd, even forms will have odd parity and vice versa. This will have a crucial impact in the partition function interpretation of quantities such as the analytic torsion and Ruelle zeta function.

### Definition 27

The assignment $(M,E)\leadsto ({\mathcal {F}}_{BF}, \Omega _{BF}, {\mathbb {S}}_{BF}, Q_{BF})$, with

59 $$\begin{aligned} Q_{BF}\mathbb {B}=d_\nabla \mathbb {B}, \qquad Q_{BF}\mathbb {A}=d_\nabla \mathbb {A}\end{aligned}$$

is called *BF* theory in the Batalin–Vilkovisky formalism.

### Analytic Torsion from Resolutions of de Rham Differential

In this section, we will discuss the relation of analytic torsion with degenerate action functionals, following Schwarz [56, 57]. This is related to BF, as we will highlight in what follows. In Sect. 4.2, we will interpret this relation in terms of a gauge fixing for *BF* theory in the Batalin–Vilkovisky framework.

Consider the geometric data $(M,E, g_M)$ of Sect. 2.2. Note that the only importance of the vector bundle is to ensure acyclicity of the twisted de Rham complex. This requirement will be necessary in Sect. 5; however, what we will say here can be extended to nontrivial cohomology along the lines of [25, 26].

We can define the partition function associated with abelian *BF* theory as follows. We first need a resolution of the kernel of the operator featuring in the action functional, represented then as the 0th cohomology of a chain complex. For abelian *BF* theory, it is given by the following:

60 $$\begin{aligned} 0\rightarrow V_{N-2}\xrightarrow {T_{N-2}} V_{N-1}\xrightarrow {T_{N-1}}\dots \xrightarrow {T_3} V_2\xrightarrow {T_2}V_1\xrightarrow {T_1}V_0\xrightarrow {T}V_0\rightarrow 0 \end{aligned}$$

where

61a $$\begin{aligned} V_0&=\Omega ^1(M)\oplus \Omega ^{N-2}(M),&T&=\begin{bmatrix} 0 &{} \star d_{N-2}\\ \star d_1 &{} 0 \end{bmatrix}; \end{aligned}$$

61b $$\begin{aligned} V_1&= \Omega ^0(M)\oplus \Omega ^{N-3}(M),&T_1&=\begin{bmatrix} d_0 &{} 0\\ 0 &{} d_{N-3} \end{bmatrix}; \end{aligned}$$

61c $$\begin{aligned} V_2&=\Omega ^{N-4}(M),&T_2&=\begin{bmatrix} 0\\ d_{N-4} \end{bmatrix}; \end{aligned}$$

61d $$\begin{aligned} V_k&=\Omega ^{N-(k+2)}(M),&T_k&=d_{N-(k+2)} \end{aligned}$$

for $3\le k\le N-2$. This implies $T_kT_k^*=d_{N-(k+2)}d_{N-(k+2)}^*$ and

62 $$\begin{aligned}&T^2=\begin{bmatrix} d_1^*d_1 &{} 0\\ 0 &{} d_{N-2}^*d_{N-2} \end{bmatrix}, \qquad T_1T_1^*=\begin{bmatrix} d_0d_0^* &{} 0\\ 0 &{} d_{N-3}d_{N-3}^* \end{bmatrix}, \qquad \nonumber \\&T_2T_2^*=\begin{bmatrix} 0 &{} 0\\ 0 &{} d_{N-4}d_{N-4}^* \end{bmatrix}. \end{aligned}$$

In fact, denoting $C:=(A,B)\in V_0$, the operator *T* allows us to rewrite

63 $$\begin{aligned} S_{BF} = \int \limits _{M} B\wedge dA = \frac{1}{2}\left( C,TC \right) \end{aligned}$$

where $(\,,\,)$ is the inner product (17) on $V_0$ as discussed in Sect. 2.2. Then, the partition function of the degenerate action functional (63), with respect to the resolution (60), is defined by Schwarz in [57] to be:

64 $$\begin{aligned} Z_{\text {Sch}}[T,T_i]:={\det }^{\flat }(T^2)^{-\frac{1}{4}}\prod _{k=1}^{N-2}{\det }^{\flat }(T_kT_k^*)^{(-1)^{k+1}\frac{1}{2}}. \end{aligned}$$

#### Remark 28

The procedure outlined above recursively produces spaces $V_k$ at every stage in order to parametrise the relevant quotients $V_k/\mathrm {ker}(T_k)$, on which integration makes sense. Indeed, said quotients coincide with $\mathrm {coker}(T_{k+1})\simeq \mathrm {ker}(T_{k+1}^*)$, and the localisation to the correct integration subspaces is obtained by restriction to $\mathrm {ker}(T^*_k)$. Thus, Schwarz’s definition of the partition function goes through an extension to a larger space of fields, and the choice of a subspace where integration is well defined: the kernel of the map ${\mathbb {T}}:=\oplus _{j=1}^{N-2}T_j^*$. Notice that this construction produces a resolution in the sense of Eq. (3).

In the case of abelian *BF* theory, this leads to

65 $$\begin{aligned} Z_{\text {Sch}}[T,T_i]= & {} {\det }^\flat (d_1^*d_1)^{-\frac{1}{4}}{\det }^\flat (d_{N-2}^*d_{N-2})^{-\frac{1}{4}}{\det }^\flat (d_0^*d_0)^{\frac{1}{2}} \nonumber \\&\times {\det }^\flat (d_{N-3}^*d_{N-3})^{\frac{1}{2}} \prod _{k=2}^{N-2}{\det }^\flat (d_{N-(k+2)}d_{N-(k+2)}^*)^{\frac{(-1)^{k+1}}{2}}.\qquad \end{aligned}$$

It is possible rewrite the expression for the partition function in a familiar form using the following lemma.

#### Lemma 29

Let $(M,E,\nabla ,\rho )$ be as in Sect. 2.1, then the following holds:

1. $${\det }^\flat (d_k^* d_k)={\det }^\flat (d_k d_k^*)$$
2. $${\det }^\flat (d_{k-1} d_{k-1}^*)= {\det }^\flat (d_{N-k}^*d_{N-k})$$
3. $${\det }^\flat (\Delta _k)={\det }^\flat (d_{k-1}d_{k-1}^*){\det }^\flat (d_{k}^*d_{k})={\det }^\flat (d_{k-1}^*d_{k-1}){\det }^\flat (d_{k}^*d_{k}).$$

#### Proof

(Sketch of proof). The proof is immediate from the analysis of the spectra of the operators under the consideration. More precisely, note that in this case ${\det }^{\flat }$s are spectral invariants as they are the usual zeta-regularised determinants.

(1) follows from the fact that the operators $d_k^*d_k$ and $d_kd_k^*$ are isospectral, a property one can check by acting with *d* on a coexact eigenform of $\Delta$.

Similarly, we observe that $$(*_{N-k})^{-1}\circ d_{k-1} d_{k-1}^*\circ *_{N-k}=d_{N-k}^*d_{N-k}$$, which implies that $$ d_{k-1} d_{k-1}^*$$ and $$d_{N-k}^*d_{N-k}$$ are isospectral and (2) follows as well.

Finally (3) follows from the observation that the spectrum of $\Delta _k=d^*_kd_k + d_{k-1}d^*_{k-1}$ is the union of spectrum of $d_{k-1}d_{k-1}^*$ and $d_k^*d_k$. $\square$

Thanks to the previous lemma, we get:

#### Proposition 30

The partition function $Z_{\text {Sch}}[T,T_i]$ for the resolution (60) yields

66 $$\begin{aligned} Z_{\text {Sch}}[T,T_i]=\tau _\rho (M). \end{aligned}$$

#### Proof

From (1) and (2) of Lemma 29, we have

$$\begin{aligned} {\det }^\flat \left( d_{N-(k+2)}d_{N-(k+2)}^*\right) ={\det }^\flat (d_{k+1}^*d_{k+1}). \end{aligned}$$

Using this relation and Lemma 29 again, we can rewrite (65) as

67 $$\begin{aligned}&Z_{\text {Sch}}[T,T_i]= {\det }^\flat (d_1^*d_1)^{-\frac{1}{4}}{\det }^\flat (d_{N-2}^*d_{N-2})^{-\frac{1}{4}}{\det }^\flat (d_0^*d_0)^{\frac{1}{2}} \nonumber \\&{\det }^\flat (d_{N-3}^*d_{N-3})^{\frac{1}{2}} \prod _{k=2}^{N-2}{\det }^\flat (d_{k+1}^*d_{k+1})^{\frac{(-1)^{k+1}}{2}}. \end{aligned}$$

Now, we know again by Lemma 29 that ${\det }^\flat (d_1^*d_1)={\det }^\flat (d_{N-2}^*d_{N-2})$ and ${\det }^\flat (d_{N-3}^*d_{N-3})={\det }^\flat (d_{2}^*d_{2}).$

Finally, using (3) of the Lemma 29, we can write

$$\begin{aligned}\prod _{k=0}^{N-1}{\det }^\flat (d_k^*d_k)^{(-1)^{k}\frac{1}{2}}= \prod _{k=1}^N{\det }^\flat (\Delta _k)^{(-1)^{k+1}\frac{k}{2}} \end{aligned}$$

In summary, we have

$$\begin{aligned} Z_{\text {Sch}}[T,T_i]=\prod _{k=1}^N{\det }^\flat (\Delta _k)^{(-1)^{k+1}\frac{k}{2}}, \end{aligned}$$

showing that the partition function $Z_{\text {Sch}}[T,T_i]$ for the resolution (60) of the operator *d* coincides with the analytic torsion $\tau _\rho (M)$ of Definition 13. $\square$

### BV Interpretation: Metric Gauge for *BF* Theory

We would like to phrase the procedure outlined in Sect. 4.1 in the Batalin–Vilkovisky framework.

First of all, we need to observe that, when the complex is acyclic, the submanifold

68 $$\begin{aligned} {\mathbb {L}}_g:\{d_\nabla ^{\star } \mathbb {B}= d_\nabla ^{\star }\mathbb {A}=0\} \end{aligned}$$

is Lagrangian in the space of BV fields ${\mathcal {F}}_{BF}$ (see Remark 5), and can be therefore used as a gauge fixing for *BF* theory. This is a consequence of Hodge decomposition (on closed manifolds without boundary). In fact, one can easily check that the images of *d* and $d^*$ are both isotropic in ${\mathcal {F}}_{BF}$, and complementary due to Hodge decomposition. We refer to this gauge fixing as the metric gauge.

Due to the vanishing of the cohomology of $\Omega ^\bullet (M,E)$, the metric gauge Lagrangian submanifold ${\mathbb {L}}_g$ can be parametrised as follows. The condition $d_\nabla ^*\mathbb {B}=0$ can be equivalently written as $\mathbb {B}=d_{\nabla }^* \eta$, and if we specify the form degree of the various fields with a subscript, we write $\mathbb {B}_{N-k-1}=d_\nabla ^* \eta _{N-k}$. Finally, writing $\eta _{N-k}=\star \tau _{k}$, together with the inner product given in Eq. 17 provides the following formula:

69 $$\begin{aligned} {\mathbb {S}}_{BF}\vert _{\mathbb {L}_g}&\equiv \int \limits _{M} \left[ \mathbb {B}d_\nabla \mathbb {A}\right] ^{\mathrm {top}}_{{\mathbb {L}}_g}\nonumber \\&=\sum _{k=0}^{N-1}\int \limits _{M}\star d_\nabla \tau _k d_\nabla \mathbb {A}_k\vert _{d^*\mathbb {A}=0} = \sum _{k=1}^N \left( \tau _k, d_\nabla ^* d_\nabla \vert _{\text {coexact}} \mathbb {A}_k\right) . \end{aligned}$$

#### Remark 31

We consider an interpretation of Eq. 66 in terms of the BV construction we just outlined.^12^ We will see how the analytic torsion can be seen as a way of making sense of the (super)determinant of the (graded) operator $d:\Omega ^\bullet _{\text {coexact}} \rightarrow \Omega ^{\bullet +1}_{\text {exact}}$. Let us define first

70 $$\begin{aligned} {\mathrm {sdet}}^\flat (d_\nabla ^*) \equiv {\mathrm {sdet}}^\flat (d_\nabla ) :={\mathrm {sdet}}^\flat (d_\nabla ^*d_\nabla )^{\frac{1}{2}}. \end{aligned}$$

Definition 9 interprets partition functions of quadratic functionals in terms of the superdeterminant of the operator that features in the functional. However, when an explicit gauge fixing is considered, one needs to take into account the parametrisation of the gauge-fixing Lagrangian. In our case this introduces a “Jacobian superdeterminant” for the change of coordinates $\mathbb {B}=d_\nabla ^*\eta$. Moreover, one should pay attention to the shift in degree $\Omega ^{-\bullet }(M,E)[1]$ and $\Omega ^{-\bullet }(M,E)[N-2]$. As a matter of fact, in this circumstance the change of variables operator $d^*$ is acting on *k*-forms of parity $k+1 \mod 2$ (instead of $k\mod 2$), so that the Jacobian superdeterminant will appear with the “opposite” power. For the same reason, recalling Remark 12, the output of a Gaussian integration over a 1-shifted graded vector space will yield the superdeterminant of the relevant operator (instead of its inverse).

Hence, one recovers the analytic torsion formally as a Gaussian integral on an supervector space by defining the partition function for gauge-fixed *BF* theory to be a flat-regularised Berezinian/superdeterminant, times the Jacobian (super)determinant $\mathrm {sdet}^\flat (d^*)^{-1}$ of the change of variables operator:

71a $$\begin{aligned} Z({\mathcal {S}}_{BF},\,{\mathbb {L}}_g)&= {\int \limits _{{\mathbb {L}}_g} \mathrm{e}^{i{\mathbb {S}}_{BF}}\vert _{{\mathbb {L}}_g} = |{\mathrm {sdet}}^\flat (d_\nabla ^*)|^{-1}\int \exp {\left( i\sum _{k=0}^{N-1} \left( \tau _{k}, (d_{k}^* d_{k})\mathbb {A}_{k}\right) \right) }} \end{aligned}$$

71b $$\begin{aligned}&:= |{\mathrm {sdet}}^\flat (d_\nabla )|^{-1} \left| {\mathrm {sdet}}^{\flat }(d_\nabla ^* d_\nabla )\right| = \left| {\mathrm {sdet}}^{\flat }(d_\nabla ^*d_\nabla )\right| ^{\frac{1}{2}}\end{aligned}$$

71c $$\begin{aligned}&= \prod _{k=0}^{N-1}{\det }^\flat (d_k^*d_k)^{(-1)^{k}\frac{1}{2}} = \tau _\rho (M), \end{aligned}$$

which, comparing with (70), means (cf. [26, Lemma 5.5])

$$\begin{aligned} {\mathrm {sdet}}^\flat (d_\nabla )\equiv {\mathrm {sdet}}^\flat (d_{\nabla }^*) := \tau _\rho (M). \end{aligned}$$

Observe that, as mentioned in Remark 10, our definition of partition function discards a potential phase factor in Eq. (70), which depends on the signature of the operator. However, as observed in [26, Proposition 5.7 and Remarks 5.8, 5.9], in the case of acyclic complexes the phase drops out. The analysis of the partition function of *BF* theory in the metric gauge fixing is discussed in detail in [26], where *BF* theory is cast on triangulated manifolds with boundary (cellular decompositions of cobordisms) and its partition function is shown to coincide with the Reidermeister torsion. The triangulation of a manifold can also be seen as a way of regularising the infinite-dimensional BV Laplacian $\Delta _{BV}$, of which ${\mathbb {S}}_{BF}$ is a cocycle.

#### Remark 32

In Schwarz’s formalism, the computation of the partition function is done by performing integration over $\mathrm {ker}({\mathbb {T}})\subset \bigoplus _{j=0}^{N-2}V_j$ (see Remark 28). It is tantamount to the 0-th Koszul–Tate cohomology, i.e. the resolution presented in Eq. (3). This can be compared with the metric gauge in the BV interpretation, where ${\mathcal {F}}_{BF}\simeq T^*[-1]\bigoplus _{k=0}^{N-2}V_k[k]$. In order to represent integration over $\mathrm {ker}({\mathbb {T}})$ within the BV formalism, one considers ${\mathbb {L}}_g$, which is essentially the same condition—i.e. restriction to coclosed forms—extended to the shifted cotangent bundle.

### Contact Gauge Fixing for *BF* Theory

This subsection shows that if *BF* theory is cast on a manifold that admits a contact structure, it is possible to find an alternative gauge-fixing condition. Adopting the same point of view on the partition function, we show how one recovers the Ruelle zeta function.

Consider the geometric data^13^ from Sect. 2.3, (*M*, *E*, *X*), and consider *BF* theory cast in the Batalin–Vilkovisky formalism as in Definition 27.

#### Proposition 33

The submanifold

$$\begin{aligned} \mathbb {L}_X:=\{(\mathbb {A},\mathbb {B})\in {\mathcal {F}}_{BF}\ |\ \iota _X\mathbb {B}=0;\, \iota _X\mathbb {A}=0\} \end{aligned}$$

is Lagrangian in ${\mathcal {F}}_{BF}$.

#### Proof

We show that the condition $\mathbb {L}_X: \iota _X\mathbb {B}=\iota _X\mathbb {A}=0$ defines an isotropic submanifold with an isotropic complement. Indeed, in virtue of the splitting in Eq. (27), we can decompose $\Omega ^\bullet (M)=\mathrm {ker}(\iota _X) \oplus \mathrm {ker}(\alpha \wedge )$, so that $\mathbb {A}=\varphi + \alpha \wedge \eta$ and $\mathbb {B}=\psi + \alpha \wedge \xi$. We observe that $\eta =\xi =0$ on $\mathbb {L}_X$, and defining $\mathbb {L}_X^\perp :=\{(\mathbb {A},\mathbb {B})\in {\mathcal {F}}_{BF}\ |\ \alpha \wedge \mathbb {A}= \alpha \wedge \mathbb {B}=0\}$, also that $\psi =\phi =0$ on $\mathbb {L}_X^\perp$. Then we have (we understand the top-form part of all the integrands)

72 $$\begin{aligned} \Omega _{BF}\vert _{\mathbb {L}_X}&= \int \limits _M \left[ \delta \psi \delta (\alpha \wedge \eta ) + \delta (\alpha \wedge \xi )\delta \varphi \right] _{\mathbb {L}_X} \equiv 0 \end{aligned}$$

73 $$\begin{aligned} \Omega _{BF}\vert _{\mathbb {L}_X^\perp }&= \int \limits _M \left[ \delta \psi \delta (\alpha \wedge \eta ) + \delta (\alpha \wedge \xi )\delta \varphi \right] _{\mathbb {L}_X^\perp } \equiv 0. \end{aligned}$$

This shows that both $\mathbb {L}_X$ and $\mathbb {L}_X^\perp$ are isotropic. $\square$

#### Definition 34

We shall refer to the choice of gauge fixing proposed in Proposition 33 for *BF* theory as the contact gauge.

#### Theorem 35

Consider the geometric data (*M*, *E*, *X*). Suppose that the Ruelle zeta function for *X* has a meromorphic extension which does not vanish at zero. Then, the Ruelle zeta function at zero $\zeta _\rho (0)$ computes the partition function of *BF* theory in the contact gauge:

74 $$\begin{aligned} Z({\mathcal {S}}_{BF},\,{\mathbb {L}}_X) = |\zeta _\rho (0)|^{(-1)^{n+1}}. \end{aligned}$$

#### Proof

We want to compute $Z({\mathcal {S}}_{BF},\,{\mathbb {L}}_X)\equiv Z({\mathbb {S}}_{BF}\vert _{\mathbb {L}_X})$. Because of the decomposition (27), we have that $\iota _X\mathbb {B}=0 \iff \mathbb {B}=(-1)^{|{\tau }|+1}\iota _X{\tau }$, for some ${\tau }$; hence,

75 $$\begin{aligned} {\mathbb {S}}_{BF}\vert _{{\mathbb {L}}_X}&= \int \limits _{M}[\mathbb {B}d_\nabla \mathbb {A}]^{\mathrm {top}}_{\mathbb {L}_X} = \int \limits _{M}[(-1)^{|{\tau }|}\iota _X{\tau } d_\nabla \mathbb {A}]^{\mathrm {top}}_{\iota _X\mathbb {A}= 0} \end{aligned}$$

76 $$\begin{aligned}&= \int \limits _M [\tau \iota _X d_\nabla \mathbb {A}]_{\iota _X\mathbb {A}=0}^{\mathrm {top}} = \int \limits _{M}[{\tau } \mathcal {L}_X\vert _{\Omega ^\bullet _0}\mathbb {A}] \end{aligned}$$

Observe that ${\tau }$ and $\mathbb {A}$ are inhomogeneous forms in $\alpha \wedge \Omega _0^\bullet (M,E)[N-2]$ and $\Omega _0^\bullet (M,E)[1]$, respectively. Then, according to Definition 9, recalling that $\mathcal {L}_{X}$ acts on a 1-shifted graded vector space and referring to Definition 14 for ${\mathrm {sdet}}^\flat (\iota _X)$, we have

77 $$\begin{aligned} Z({\mathcal {S}}_{BF},\,{\mathbb {L}}_X) = \left| \mathrm {sdet}^\flat \mathcal {L}_{X}\vert _{\Omega ^\bullet _0}\right| = \left| \prod _{k=0}^{N-1} \left( {\det }^\flat \mathcal {L}_{X,k}\vert _{\Omega ^k_0}\right) ^{(-1)^k}\right| . \end{aligned}$$

This, compared with Eqs. (40) and (50), allows us to conclude the proof. $\square$

#### Remark 36

We would like to stress how the crucial element in both metric and contact gauges is the existence of a “Hodge decomposition” for the space of *k*-forms. In the metric case, it is given, in particular, by the kernel of the de Rham differential and its dual, but truly it can be considered independently of it, as in the contact case, where the maps $\iota _X$ and $\alpha \wedge$ define the splitting.

#### Remark 37

It is tempting to consider a field theory with action functional $S_X:=\int _{M} [\phi \mathcal {L}_X \psi ]^{\text {top}}$ on differential forms $\phi ,\psi \in \Omega ^\bullet (M)$, and call it Ruelle theory. Observe that this is what one gets out of Theorem 35, but with a nontrivial shift in degree. This is akin to considering the theory $\int _{M}[{\mathcal {B}}d{\mathcal {A}}]^{\text {top}}$, for unshifted inhomogeneous differential forms ${\mathcal {A}},{\mathcal {B}}\in \Omega ^\bullet$, but—to the best of our knowledge—that does not seem to have a clear interpretation up to now.

## Lagrangian Homotopies, Fried’s Conjecture and Gauge-fixing Independence

In this section, we outline a strategy to interpret the recent results of [32] on Fried’s conjecture as invariance of gauge fixing in the BV formalism, and vice versa. We will set up a general geometric framework to discuss perturbation of the Anosov vector field as an argument for the Ruelle zeta function, and argue how this can be used to construct homotopies for Lagrangian submanifolds. Indeed, consider the following:

### Claim 38

Assume Theorem 2 holds on ${\mathcal {F}}_{BF}$ for some appropriate choice of BV Laplacian $\Delta _{BV}$, and assume there exists a Lagrangian homotopy between ${\mathbb {L}}_X$ and ${\mathbb {L}}_g$ for any *X* contact and Anosov. Then, up to phase,

78 $$\begin{aligned} \tau _\rho (M) = Z({\mathcal {S}}_{BF},\,{\mathbb {L}}_g) = Z({\mathcal {S}}_{BF},\,{\mathbb {L}}_X) = |\zeta _\rho (0)|^{(-1)^{n}}, \end{aligned}$$

which is the statement of (Fried’s) Conjecture 21.

### Remark 39

We stress here that a universal generalisation of Theorem 2 to infinite dimensions, i.e. one that works independently of the details of the field theory (and gauge fixing), is currently not yet available. The results of [26] suggest that one possible regularisation scheme for the BV Laplacian might arise as a limit of finite-dimensional cellular analogues. The works of [27] and [40, 44] assume instead a somewhat different set-up, which would also need to be adapted to the case at hand. Ideally, a scheme should be found that makes the regularisation of the BV Laplacian compatible with the context of Definition 7. Observe that the recent results of [20] suggest that a number of subtleties might arise, already in acyclic cases.

The central notions for this section, and the main theorem we will need are as follows.

### Definition 40

Let *X* be an Anosov vector field on a manifold *M*. We will say that *X* is regular whenever the restricted resolvents $R_k(\lambda )=(\lambda _{X,k} + \lambda )^{-1}$ of Eq. (52) do not have poles at $\lambda =0$ for all $0\le k \le N-1$.

Recalling Remark 22, we report now a result by Dang, Guillarmou, Rivière and Shen on the properties of Ruelle zeta function seen as a function on Anosov vector fields:

### Theorem 41

([32]). Let (*M*, *E*) denote the geometric data of Sect. 2.1. Consider the set $U\subset C^\infty (M,TM)$ of regular smooth Anosov vector fields *X*. Then this set is open, and the map $\zeta :U \rightarrow {\mathbb {C}}$, sending *X* to $\zeta (X,0)$, is locally constant and nonzero.

### A Sphere Bundle Construction

Let $\Sigma$ be a compact manifold. We denote by ${\mathcal {R}}(\Sigma )$ the space of all Riemannian metrics on $\Sigma$, by ${\mathcal {R}}_{<}(\Sigma )$ the space of negative sectional curvature metrics, and by ${\mathcal {R}}_h(\Sigma )$ the space of hyperbolic metrics.

#### Definition 42

Let us fix a reference metric $g_0$. We denote the sphere bundle associated with $g_0$ by $S_0^*\Sigma :=S_{g_0}^*\Sigma$. Moreover, let $g,{\widetilde{g}}\in {\mathcal {R}}(\Sigma )$ and consider the diffeomorphism

79 $$\begin{aligned} \sigma _g^{{\widetilde{g}}}:S_g^*\Sigma \longrightarrow S_{{\widetilde{g}}}^*\Sigma \end{aligned}$$

obtained by rescaling lengths in $T\Sigma$. Then, we denote the diffeomorphisms $\sigma _g:S_g^*\Sigma \rightarrow S_0^*\Sigma$, with $\sigma _g:=\sigma ^{g_0}_g$ for all $g\in {\mathcal {R}}(\Sigma )$. Denoting by $\varphi _g$ the geodesic flow on $S_g^*\Sigma$, and by $X_g$ the associated vector field. One can transfer the geodesic flow $\varphi _g$ to $S_0^*\Sigma$ by $\varphi _g^0=\sigma _g \circ \varphi _g \circ \sigma _g^{-1}$; we denote the associated vector field on $S_0^*\Sigma$ by $X_g^0$.

Since we are interested in computing the Ruelle zeta function on sphere bundles, we would like to ensure that the geodesic (Reeb) vector field $X_g$ is Anosov in $S_g^*\Sigma$. This would not be true in general, but it will be true for metrics of negative sectional curvature ${\mathcal {R}}_<$. The map $\sigma _g$ allows us to consider all Anosov geodesic vector fields on the same reference space $S_0^*\Sigma$.

#### Lemma 43

The Anosov property of is preserved under pushforward by $\sigma _g$.

#### Proof

This is an adaptation of a result proved in [49]. Given $S_g^*\Sigma$ with Anosov flow $\varphi _g$ and associated vector field $X_g$, we also have a decomposition

80 $$\begin{aligned} T(S_g^*\Sigma ) = \mathbb {R}X_g + E_s(X_g) + E_u(X_g) \end{aligned}$$

with constants $C_g, \lambda _g$ so that for all $v_s\in E_s(X_g)$, $v_u\in E_u(X_g)$ and all $t>0$:

81 $$\begin{aligned} \Vert \mathrm{d}\varphi _{g,t} v_s \Vert _g \le C_g \mathrm{e}^{-\lambda _g t} \Vert v_s \Vert _g, \qquad \Vert \mathrm{d}\varphi _{g,-t} v_u \Vert _g \le C_g \mathrm{e}^{-\lambda _g t} \Vert v_u \Vert _g. \end{aligned}$$

Using $\mathrm{d}\sigma _g$, we push the decomposition of $T(S_g^*\Sigma )$ to $T(S_0^*\Sigma )$ providing

82 $$\begin{aligned} T(S_0^*\Sigma ) = \mathbb {R}X_g^0 + E_s(X_g^0) + E_u(X_g^0) \end{aligned}$$

where $E_s(X_g^0) :=\mathrm{d}\sigma _g (E_s(X_g))$ and $E_u(X_g^0) :=\mathrm{d}\sigma _g (E_u(X_g))$. Now $\mathrm{d}\sigma _g$ and $\mathrm{d}\sigma _g^{-1}$ are both bounded so for $v_s^0\in E_s(X_g^0)$, there is $v_s \in E_s$ such that $\mathrm{d}\sigma _g v_s = v_s^0$, whence for $t>0$:

83 $$\begin{aligned}&\Vert \mathrm{d}\varphi _{g,t}^0 v_s^0\Vert _0 \nonumber \\&\quad =\Vert \mathrm{d}\sigma _g \mathrm{d}\varphi _{g,t} v_s \Vert _0 \le \Vert \mathrm{d}\sigma _g\Vert \Vert \mathrm{d}\varphi _{g,t} v_s \Vert _g \le \Vert \mathrm{d}\sigma _g\Vert C_g \mathrm{e}^{-\lambda _g t} \Vert v_s\Vert _g \end{aligned}$$

84 $$\begin{aligned}&\quad = \Vert \mathrm{d}\sigma _g\Vert C_g \mathrm{e}^{-\lambda _g t} \Vert \mathrm{d}\sigma _g^{-1} v_s^0\Vert _g \le \Vert \mathrm{d}\sigma _g\Vert \Vert \mathrm{d}\sigma _g^{-1}\Vert C_g \mathrm{e}^{-\lambda _g t} \Vert v_s^0\Vert _0 = C_g' \mathrm{e}^{-\lambda _g t} \Vert v_s^0\Vert _0. \end{aligned}$$

A similar result holds for vectors in $E_u(X_g^0)$. $\square$

#### Definition 44

We define the assignment:

85 $$\begin{aligned} {\mathbb {X}} :{\mathcal {R}}(\Sigma ) \longrightarrow C^\infty (S_0^*\Sigma ; T(S_0^*\Sigma )); \qquad {\mathbb {X}}(g)=\mathrm{d}\sigma _g X_g=X_g^0 \end{aligned}$$

where $X_g$ is the geodesic vector field induced by *g* on $S_g^*\Sigma$.

#### Proposition 45

Let us denote by ${\mathcal {A}}(\Sigma )$ the set of Anosov vector fields on $S_0^*\Sigma$, and by ${\mathcal {R}}_<(\Sigma )$ the space of negative sectional curvature metrics on $\Sigma$. Then

86 $$\begin{aligned} {\mathcal {A}}_<(\Sigma ) :=\mathrm {Im}\left( {\mathbb {X}}\vert _{{\mathcal {R}}_<(\Sigma )}\right) \subset {\mathcal {A}}(\Sigma ). \end{aligned}$$

#### Proof

This follows from the general fact that all negative sectional curvature metrics have Anosov geodesic flows, and application of Lemma 43. $\square$

#### Lemma 46

For $\Sigma$ a two-dimensional surface of negative Euler characteristic $\chi (\Sigma )<0$, the image of ${\mathbb {X}}$ restricted to ${\mathcal {R}}_h(\Sigma )$, the space of hyperbolic metrics on $\Sigma$, is a deformation retract of ${\mathcal {A}}_{<}(\Sigma )$.

#### Proof

On two-dimensional surfaces, the space of negative sectional curvature metrics coincides with the space of negative Ricci curvature metrics, which is a contractible subset of ${\mathcal {R}}$. In fact, via the Ricci flow one canonically deforms any metric *g* into a hyperbolic one, and in particular, negatively curved metrics are deformed in such a way along a path of negatively curved metrics [60]. Thus, the space of hyperbolic metrics on $\Sigma$ is a deformation retract of the space of all negatively curved metrics ${\mathcal {R}}_<(\Sigma )$, and so will be its image under ${\mathbb {X}}$ with respect to the space ${\mathcal {A}}_{<}(\Sigma )$. $\square$

#### Theorem 47

Consider the contact Anosov–Riemannian structure $(M, E, X_g, g)$ of Sect. 2.4.1, where $M=S^*_g\Sigma$ with $\mathrm {dim}(\Sigma )=2$, the Euler characteristic $\chi (\Sigma )<0$, and $g\in {\mathcal {R}}_<(\Sigma )$. Then,

87 $$\begin{aligned} |\zeta (X_g,0)|=\tau _\rho (M)^{-1}. \end{aligned}$$

#### Proof

In virtue of Lemma 46, there exists a smooth path *X*(*t*) of Anosov vector fields in ${\mathcal {A}}_<(\Sigma )$ connecting $X_g$ to $X_{g_h}$ for some $g_h$ hyperbolic, where Theorem 20 holds: let *g*(*t*) be a retraction such that $g(0)=g_0$ and $g(1)=g$, and set $X(t)={\mathbb {X}}(g(t))$. If we can show that each *X*(*t*) is regular, then local constancy of the zeta function at zero (Theorem 41) provides the result.

The idea for showing regularity is essentially in [34] even though that setting is using the trivial representation, so that the de Rham complex is not acyclic. A more explicit version which adapts the spirit of [34] is in [32]. Below we cite the necessary results of [32] to conclude that *X*(*t*) is regular.

Since $g\in {\mathcal {R}}_<(\Sigma )$, its associated geodesic vector field $X_g$ on $S_g^*\Sigma$ is Anosov [1, 5, 6]. For regularity, one shows that no *k*-form resonant states $\varphi ^{(k)}$ associated with the spectral parameter $\lambda =0$ exist for $k\in \{0,1,2\}$ (recall Eq. (54)). By [32, Lemma 7.4], 0-form resonant states $\varphi ^{(0)}$ are closed and smooth in this setting so may be identified with degree-0 de Rham cohomology. Acyclicity implies such states are necessarily the 0-section. For 2-form resonant states $\varphi ^{(k)}$, there is an isomorphism with 0-form resonant states upon wedging with $\mathrm{d}\alpha$ [32, Lemma 7.2]. Effectively $\varphi ^{(2)}=\varphi ^{(0)}\mathrm{d}\alpha$ so in this case also, no such resonant states exist. Finally, for 1-form resonant states, we require degree-1 cohomology to vanish and appeal to [32, Lemma 7.2 Hypothesis (1)] to conclude no nontrivial resonant states exist. $\square$

#### Corollary 48

Under the assumptions of Theorem 47, denoting by

$$\begin{aligned} {\mathbb {L}}_X=\{(\mathbb {A},\mathbb {B})\in {\mathcal {F}}_{BF}\ |\ \iota _{{\mathbb {X}}(g)}\mathbb {A}=\iota _{{\mathbb {X}}(g)}\mathbb {B}=0\} \end{aligned}$$

the Lagrangian submanifold defined by the Anosov vector field ${\mathbb {X}}(g)$, we have

88 $$\begin{aligned} Z({\mathcal {S}}_{BF},\,{\mathbb {L}}_X)=\tau _\rho (M)^{-1}, \end{aligned}$$

for every $$g\in {\mathcal {R}}_<(\Sigma )$$.

#### Proof

This follows from Theorems 35 and 47. $\square$

#### Remark 49

Observe that the above construction can be generalised to some extent. In general, the space of negative sectional curvature metric will not be path connected, so let us consider the connected components that contain a hyperbolic metric. The image under ${\mathbb {X}}$ of the disjoint union of such connected components in ${\mathcal {A}}(\Sigma )$ will be a union of islands of Anosov vector fields, path connected to an Anosov vector fields in $\mathrm {Im}({\mathbb {X}}\vert _{{\mathcal {R}}_h})$. In a neighbourhood of a hyperbolic metric Anosov vector field, the requirements of Theorem 41 are satisfied, and Fried’s conjecture might be extended to open subsets of ${\mathcal {A}}_<(\Sigma )$. We defer the development of such a generalisation to a subsequent work.

#### Remark 50

The spirit of gauge-fixing homotopies is that of replacing an ill-defined integral with a well-defined one, and can be considered as providing a family of integral representations of the same quantity, only some of which are directly computable. From this point of view, the Ruelle zeta function at zero might not be computable for a generic (Anosov) vector field, but it will be once deformed away from an invalid point in ${\mathcal {A}}(\Sigma )$.

We wish to interpret this result in terms of homotopies of Lagrangian submanifolds in ${\mathcal {F}}_{BF}$ and gauge-fixing independence for *BF* theory.

#### Theorem 51

Consider the geometric data $(M, E, X_g, g)$ of Sect. 2.4.1, a smooth path $g_t: [0,1] \rightarrow {\mathcal {R}}(\Sigma )$ such that ${\mathbb {X}}(g_0)$ is regular, and let

$$\begin{aligned} \mathbb {L}_t:=\{(\mathbb {A},\mathbb {B})\in {\mathcal {F}}_{BF}\ |\ \iota _{{\mathbb {X}}(g_t)}\mathbb {A}=\iota _{{\mathbb {X}}(g_t)}\mathbb {B}=0\} \end{aligned}$$

be the associated smooth family of Lagrangian submanifolds in ${\mathcal {F}}_{BF}$. Then, $Z({\mathcal {S}}_{BF},\,{\mathbb {L}}_0)\not = 0$ and

89 $$\begin{aligned} \frac{d}{\mathrm{d}t}\Big \vert _{t=0} Z({\mathcal {S}}_{BF},\,{\mathbb {L}}_t) =0. \end{aligned}$$

Moreover, if $g_0$ is hyperbolic, we have that

90 $$\begin{aligned} Z({\mathcal {S}}_{BF},\,{\mathbb {L}}_0) = \tau _\rho (M). \end{aligned}$$

#### Proof

Lemma 43 ensures that ${\mathbb {X}}(g_t)=\mathrm{d}\sigma _{g_t}X_{g_t}$ is a smooth path of Anosov vector fields in $S_0^*\Sigma$, and in virtue of Theorem 35, we have that

$$\begin{aligned} Z({\mathcal {S}}_{BF},\,{\mathbb {L}}_t) \equiv Z({\mathbb {S}}_R\vert _{{\mathbb {L}}_t}) = |\zeta ({\mathbb {X}}(g_t),0)|^{(-1)^n}. \end{aligned}$$

By assumption, at $g_0$ the *k*th Ruelle zeta factors $\zeta _k({\mathbb {X}}(g_0),0)$ are well defined and different from zero. Then, in virtue of Theorem 41$\zeta ({\mathbb {X}}(g_0),0)$ is constant in a open neighbourhood of ${\mathbb {X}}(g_0)$; hence, it is on the whole path $g_t$ for $t\in [0,T)$ for some appropriately chosen *T*, and in particular, its derivative at *t* vanishes. If we choose $g_0$ hyperbolic, using Theorem 20 we can conclude that

$$\begin{aligned} Z({\mathcal {S}}_{BF},\,{\mathbb {L}}_0) = |\zeta ({\mathbb {X}}(g_0),0)|^{(-1)^n} \equiv |\zeta _{\rho }(M)|^{(-1)^n}= \tau _\rho (M). \end{aligned}$$

$$\square $$

## Footnotes

1 This is often called Lorenz gauge fixing.

2 More generally, a sheaf.

3 There is an equivalent formulation of this in the variational bi-complex [4, 31], where the full jet bundle is taken into account.

4 We will only be concerned with continuous symmetries.

5 In some practical cases, the vanishing of the negative cohomology is not guaranteed. We will anyway not need this condition in what follows.

6 *Q* is essentially the derivation $d_C$ interpreted as a vector field.

7 This notion coincides with requiring *L* to be maximal isotropic.

8 Observe that we are considering parities modulo 2. In principle, a graded determinant would return $\prod _{k=0}^n \det (A)^{k(-1)^k}$. We will not make such a distinction in what follows.

9 Note that the $V'$ components in *V* are not considered to have different degrees, i.e. *A* is an even matrix of graded operators.

10 The name *BF* comes from the tradition of denoting fields with *B* and *A*, and the Lagrangian density with $B\wedge F_A$.

11 Strictly speaking this is true only up to boundary terms. We will assume that *M* has no boundary, but otherwise the analysis of boundary terms is relevant, and can be performed with a version of the BV formalism [25].

12 The authors would like to thank P. Mnev and A.S. Cattaneo for valuable insight into this interpretation.

13 Again, the importance of *E* is to ensure acyclicity.
